# Identification of *Escherichia coli ygaQ* and *rpmG* as novel mitomycin C resistance factors implicated in DNA repair

**DOI:** 10.1042/BSR20150249

**Published:** 2016-01-22

**Authors:** Edward L. Bolt, Tabitha Jenkins, Valeria Moreira Russo, Sharlene Ahmed, James Cavey, Simon D. Cass

**Affiliations:** * School of Life Sciences, The University of Nottingham, Nottingham NG72UH, U.K.

**Keywords:** DNA repair, homologous recombination, mitomycin C, rpmG, ygaQ

## Abstract

Using the ASKA (A Complete Set of *Escherichia coli* K-12 ORF Archive) library for genome-wide screening of *E. coli* proteins we identified that expression of *ygaQ* and *rpmG* promotes mitomycin C resistance (MMC^R^). YgaQ mediated MMC^R^ was independent of homologous recombination involving RecA or RuvABC, but required UvrD. YgaQ is an uncharacterized protein homologous with α-amylases that we identified to have nuclease activity directed to ssDNA of 5′ flaps. Nuclease activity was inactivated by mutation of two amino acid motifs, which also abolished MMC^R^. RpmG is frequently annotated as a bacterial ribosomal protein, although forms an operon with MutM glycosylase and a putative deubiquitinating (DUB) enzyme, YicR. RpmG associated MMC^R^ was dependent on MutM. MMC^R^ from RpmG resembles DNA repair phenotypes reported for ‘idiosyncratic ribosomal proteins’ in eukaryotes.

## Abstract (precis)

A genome-wide protein expression screen in *Escherichia coli* has identified new mitomycin C resistance factors, genes *ygaQ* and *rpmG*. These were characterized, revealing that *ygaQ* encodes a new nuclease enzyme and that RpmG is likely be an “idiosyncratic ribosomal protein” with a role in DNA repair by MutM.

## INTRODUCTION

Chemicals causing covalent modifications to DNA are cytotoxic when their products interfere with biological processes including DNA replication and gene transcription. Mitomycin C (MMC) provokes interstrand DNA cross-links at 5′-GNC-3′ or 5′-CG-3′ sequences, and mono-adducts at guanine bases [1–3]. It is a natural antimicrobial synthesized by *Streptomyces caespitosis* that is effective as a treatment for human cancers, and there is continuing interest in mechanisms cells use to overcome genotoxic damage associated with MMC and other cross-linkers [4].

Removal and repair of MMC induced DNA damage, involves interplay between nucleotide excision repair, homologous recombination and repair polymerases. Recent reviews detail the multiple factors implicated in repair of DNA cross-links in human cells and in prokaryotes [4,5]. In bacteria, UvrA, UvrB and UvrC nucleotide excision repair complexes recognize and eliminate DNA-MMC lesions [5]. DNA molecules generated during and after UvrABC processing can be used as substrates for gap repair by DNA polymerase I, and for homologous recombination initiated by RecA or RecFOR and controlled and completed by helicases (RecG, RuvAB, UvrD, RecQ), and resolvases (RuvC, RecU). The exact events post-excision of the lesion probably depend on the context of repair and the type of lesion being removed.

The importance of homologous recombination for repair of MMC cross-links in *Escherichia coli* is illustrated by the high MMC sensitivity of cells lacking the Holliday junction helicase RuvAB or Holliday junction resolvase RuvC (Δ*ruvAB/*Δ*ruvC*) [6,7]. RuvAB and RuvC associate into a ‘RuvABC resolvasome’ that assists in double strand break repair by branch migrating and resolving Holliday junction DNA into nicked DNA duplexes [8–13]. Similar activities of RuvABC at blocked replication forks can promote repair of blocking lesions and restart of replication [7,14–16]. Δ*ruvABC* cells can be rescued from MMC sensitivity by expression of alternative Holliday junction nucleases, the archaeal resolvase Hjc [17], or bacteriophage RusA [18]. Deletion in *E. coli* of base excision repair (BER) enzyme MutM and nucleotide excision repair (NER) enzymes UvrABC also cause acute sensitivity to MMC [5], highlighting how multiple DNA repair roles are be needed to overcome genotoxic effects of MMC.

DNA repair has been intensively studied in *E. coli* to identify DNA repair pathways by genetic analysis, followed by detailed understanding of DNA repair enzyme structure and function [8,19–24]. DNA repair genes may remain to be identified in *E. coli*, considering the unknown roles for about 30% of *E. coli* genes. A recent genetic screen in *E. coli* unearthed and validated roles for uncharacterized genes in promoting resistance to extreme ionizing radiation [22]. Using protein expression from the ASKA (A Complete Set of *Escherichia coli* K-12 ORF Archive) genomic library [25] we screened for MMC resistance of Δ*ruvABC* cells, identifying four genes with a validated MMC^R^ phenotype. Two of these, *ygaQ* and *rpmG*, are reported in more detail here.

## RESULTS

### Identification of *ygaQ* and *rpmG* as mitomycin C resistance factors in *E. coli*

**Figure 1:**
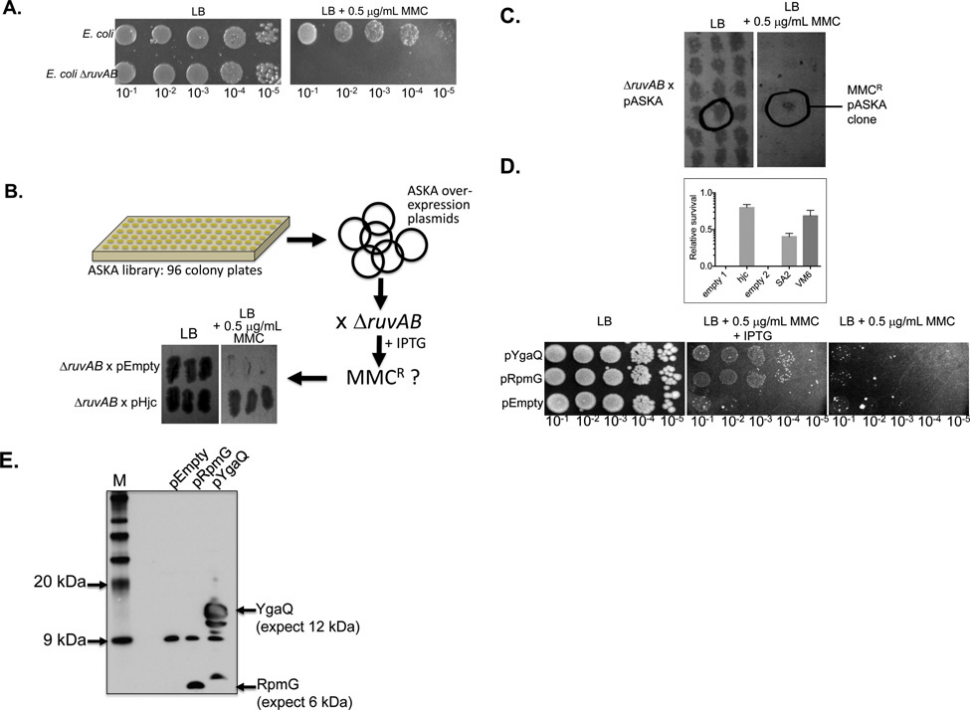
Identification and analysis of YgaQ and RpmG genes and proteins as mitomycin C resistance factors during an ASKA library screen (**A**) Viability spot test to illustrate MMC sensitivity of the *E. coli* Δ*ruvAB* strain used for screening the ASKA library for MMC^R^. (**B**) The screening procedure. Plasmid DNA isolated from combining typically 96 colonies from an individual ASKA library agar plate was transformed into *E. coli* Δ*ruvAB*. Growth of colonies after plating out on LB agar containing MMC was used to assess MMC^R^ when compared with that given by plasmid expression of Hjc resolvase as a positive control, as shown in the panel. Further experimental details, including how *ruvAB* induced false positives were avoided, are given in the methods section. (**C**) Example of a MMC^R^ clone arising from the ASKA screen. The panels show details of agar plates after gridding individual colonies in the presence or absence of MMC as indicated. (**D**) Analysis of MMC^R^ provided by expression of YgaQ or RpmG, dependent on addition of IPTG to growth media. The graph compares viable colony counts from spot tests in triplicate using Δ*ruvAB* cells transformed by either pHjc (a positive control that restores MMC^R^ (17)) and its corresponding empty vector (empty 1, pT7-7), or by ASKA plasmids (Supplementary Table S2) harbouring *rpmG* (SA2) or *ygaQ* (VM6) and its empty plasmid control (empty 2). A photograph of an example viability spot test for these clones is presented in the panels below. (**E**) Western blot of total cell protein extracted from cultures used to make the viability spot tests shown in (D). YgaQ and RpmG proteins were detected using antibody against their hexa-histidine tag.

We searched for uncharacterized genes in *E. coli* whose expression overcame growth inviability associated with MMC induced DNA damage. The genetic assay we used exploited the extreme MMC sensitivity of an *E. coli* Δ*ruvAB* strain (Figure 1A) resulting from it lacking the RuvABC DNA repair complex. This followed a rationale from previous work identifying that the archaeal Holliday junction resolvase Hjc can restore mitomycin C resistance (MMC^R^) to Δ*ruvAB* cells [17] (Figure 1B). An ASKA plasmid library [25] was transformed into *E. coli* Δ*ruvAB,* followed by viability tests on MMC agar, resulting in 21 colonies with apparent MMC^R^ compared with surrounding colonies on replica agar plates, summarized in Figure 1(C). Four of these clones were verified for MMC^R^ in multiple repeats of the same assay, judged by each growing comparably to pHjc on MMC agar (Supplementary Table S3). Two of these clones (pSTE5 and pDO4) had a strong negative fitness effect on cell viability when expressed in ‘wild type’ (RuvAB^+^) *E. coli*, and were therefore discarded from the remainder of the present study. The two other MMC^R^ ASKA clone plasmids, pSA2 and pVM6, were investigated further.

DNA sequencing confirmed that pVM6 and pSA2 contained, respectively, *E. coli* genes *ygaQ* and *rpmG*. *E. coli* Δ*ruvAB* cells expressing *ygaQ* or *rpmG* (pYgaQ/pRpmG) were 1000-fold more viable than empty plasmid control, and this effect was dependent on IPTG induction of plasmid gene expression (Figure 1D). Western blotting of proteins from the same IPTG induced MMC^R^ cultures detected proteins consistent with predicted sizes consistent with YgaQ and RpmG proteins that were absent from cells containing only empty plasmid vector (Figure 1E). YgaQ also showed multiple protein species of lower than expected molecular mass, probably representing isoforms or protein degradation. MMC^R^ of pYgaQ or pRpmG colonies remained dependent on IPTG for plasmid gene expression when sub-cultured as fresh overnight growths, confirming that chromosomal suppressors did not account for the observed phenotype. The same cultures spread on to agar containing rifampicin (0–50 μg/ml) did not show evidence of a mutator phenotype, which could promote MMC^R^ independently of pYgaQ or pRpmG, compared with wild type cells and a Δ*mutS* hyper-mutator control. YgaQ and RpmG were therefore further characterized: aside from their ability to promote MMC^R^ in *E. coli*, they have no obvious relationship to one another in genomic context or predicted protein function, as detailed below, and are therefore dealt with separately.

### Mutagenesis of YgaQ abolishes mitomycin C resistance

**Figure 2:**
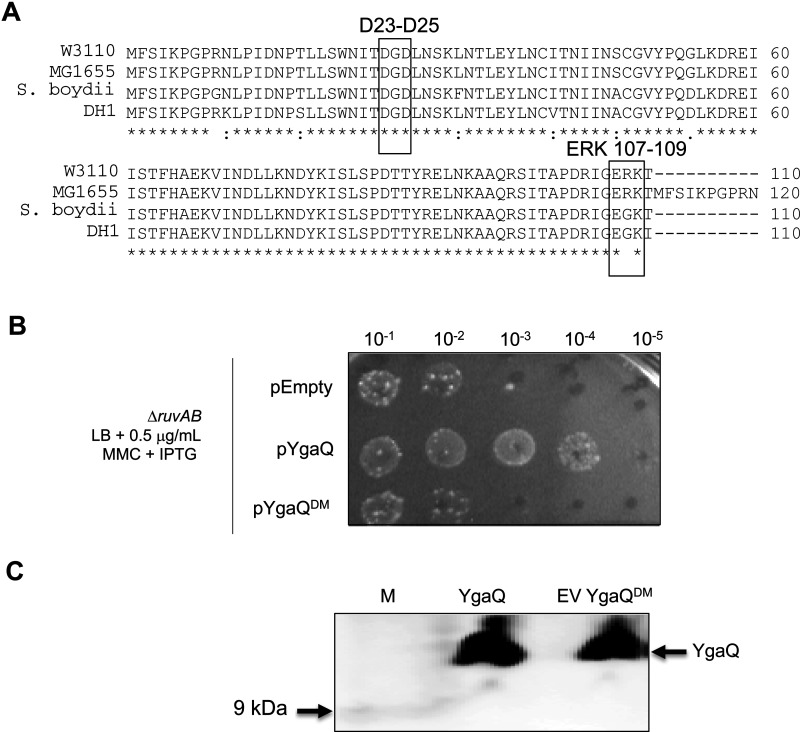
Analysis of YgaQ containing site-directed mutations (**A**) A ClustalW alignment of YgaQ amino acid sequences from *E. coli* W3110 (the strain used to make the ASKA library (25), *E. coli* MG1655, *Shigella boydii* and *E. coli* DH1. Highlighted in boxes are the two regions of W3110 YgaQ that when mutagenized in combination gave YgaQ^DM^ that could not promote MMC^R^ and gave nuclease defective YgaQ protein. (**B**) Example of a viability spot test comparing the MMC^R^ of expression from ASKA plasmids YgaQ or YgaQ^DM^. (**C**) Western blot confirming that YgaQ^DM^ protein is expressed like YgaQ in cells used for the viability spot test in (**B**).

The *ygaQ* gene of *E. coli* strain W3110, used for construction of the ASKA library, encodes an uncharacterized protein of 110 amino acids with no conserved domains. *ygaQ* is present in *Escherichia* and *Shigella* species, located next to a predicted α-amylase gene *ygaR*. In some *E. coli* strains (e.g. MG1655) it is predicted that *ygaQ* and *ygaR* are fused as a single open reading frame; more detailed analysis of YgaQ is presented in Supplementary results Figures S1 and S2. Alignment of YgaQ homologues identified many conserved amino acids in YgaQ (Figure 2A). We focused on mutagenesis of individual and combined glutamic and aspartic acid amino acid residues within pYgaQ because of their essential catalytic roles in microbial alpha-amylases. Resulting plasmids were tested for their ability to promote MMC^R^ in Δ*ruvAB* cells compared with wild type pYgaQ. Combining mutations of Asp-25 and Asp-27 with mutation of a Glu-Arg-Lys triplet at the YgaQ C-terminus (a mutant subsequently referred to as YgaQ^DM^) abolished MMC^R^, similarly to the empty plasmid control (Figure 2B). Western blotting confirmed expression YgaQ^DM^ like wild type protein (Figure 2C), confirming that protein mis-folding or instability is unlikely to explain MMC sensitivity from expressing this mutant YgaQ. Furthermore YgaQ^DM^ was expressed as soluble protein for purification, as described below. We concluded that MMC^R^ associated with YgaQ was specific to this protein, and that the mutagenized residues are important for the biological function of YgaQ when promoting MMC^R^ in cells lacking DNA repair by RuvABC.

### YgaQ MMC^R^ requires UvrD and acts independently of homologous recombination

**Figure 3:**
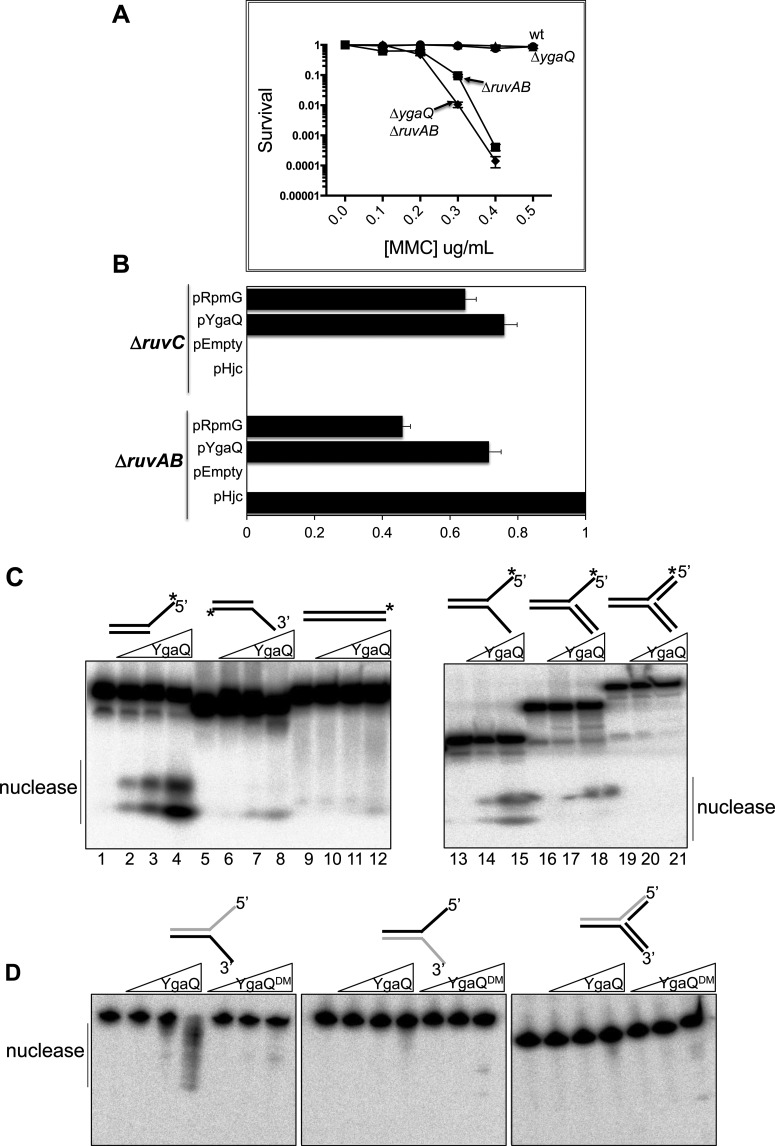
YgaQ is a nuclease that acts independently of Holliday junction processing by RuvABC (**A**) Graph ‘killing curves’ comparing strains Δ*ygaQ*, Δ*ruvAB*  and Δ*ruvAB* Δ*ygaQ* for MMC sensitivity in viability spot tests plotted as a function of MMC concentration as indicated. The assays were done in triplicate with bars representing standard error. (**B**) Graph showing survival of ASKA plasmids expressing YgaQ (pYgaQ) or RpmG (pRpmG) compared with the positive control pHjc and corresponding empty ASKA plasmid vector. Assays were done twice and standard error from the mean is given as bars. (**C**) Non-denaturing TBE acrylamide gel for analysis of products from mixing YgaQ with DNA substrates as indicated. YgaQ was used at 0, 2.5, 25 and 250 nM (lanes 1–12) or 0, 25 and 250 nM (lanes 13–21) in reactions containing 0.6 nM of DNA that was ^32^P 5′-end-labelled as indicated with (*). (**D**) Urea denaturing TBE acrylamide gels for analysis of products from mixing YgaQ with forked DNA as indicated; in each substrate the strand presented in grey is labelled at its 5′ end. YgaQ and YgaQ^DM^ mutant proteins were each used at 0, 2.5, 25 or 250 nM in reactions containing 0.6 nM of DNA.

Elimination of *ygaQ* (Δ*ygaQ*) from RuvABC^+^ *E. coli* had no effect on cell viability in MMC agar compared with YgaQ^+^ cells. Δ*ruvAB* cells were very sick as expected, but combining Δ*ygaQ* with Δ*ruvAB* caused modest, but reproducible, increased sensitivity to MMC compared with Δ*ruvAB* alone (Figure 3A). This is consistent with YgaQ acting independently of RuvABC Holliday junction processing, as expected from the original screening analysis. We returned to pYgaQ to explore this further, testing if it restored MMC^R^ to *E. coli* Δ*ruvC* cells, in which RuvAB is present. The rationale for this test is based on inability of Hjc and RusA resolvases to restore MMC^R^ to Δ*ruvC* cells, even though they rescue Δ*ruvAB* cells: access of Hjc and RusA to Holliday junctions is blocked by RuvAB. However, unlike Hjc and RusA, YgaQ restored MMC^R^ to both Δ*ruvC* cells, consistent with it not targeting Holliday junctions (Figure 3B).

We tested if pYgaQ restored MMC^R^ to Δ*ruvAB* cells that contained additional deletions of genes in DNA repair pathways: *recA,* for recombination dependent repair without Holliday junction formation*, dinG* and *umuD* for translesion synthesis*, recG and uvrD* for DNA repair linked to replication stress*,* and *uvrB* for excision repair. Interestingly, only deletion of *uvrD* (therefore Δ*ruvAB* Δ*uvrD*) caused pYgaQ to be unable to restore MMC^R^ (Supplementary Figure S3). These data indicate that MMC^R^ from YgaQ expression is independent of homologous recombination, and that it might participate in UvrD driven DNA repair processes at blocked replication forks [26]. We purified *E. coli* YgaQ proteins to assay for DNA binding and catalytic activities to gain more understanding of involvement in MMC^R^.

### Purified YgaQ protein is a nuclease that targets single-stranded DNA

*E. coli* strain W3110 YgaQ and YgaQ^DM^ proteins were purified (Supplementary Figure S4) and assayed *in vitro* for DNA binding and processing of branched DNA substrates that mimic intermediates formed during DNA repair, replication and recombination. EMSAs mixing purified YgaQ with DNA substrates were inconclusive in determining any substrate binding specificity because YgaQ repeatedly formed in-well aggregates rather than binding complexes. However, YgaQ catalytic activity was identified in similar reactions supplemented with 10 mM Mg^2+^ and stopped by treating with proteinase K prior to electrophoresis (Figures 3C and 3D). Native gels showed YgaQ dependent product formation consistent with nuclease activity on partial and flayed duplex substrates containing ssDNA with a 5′ end (Figure 3C). Fully base paired DNA substrates, or substrates with ssDNA terminating at a 3′OH, gave very weak or no activity. A preference for YgaQ targeting 5′-ended ssDNA was confirmed using denaturing gels (Figure 3D); nuclease activity was detected on ssDNA with 5′-terminus, but not on the strand with opposite polarity terminating in 3′OH. No activity was detected on the same strand in a fully based paired fork, confirming that YgaQ targets ssDNA. In the same assays YgaQ^DM^ showed greatly reduced activity, in agreement with loss of the MMC^R^ phenotype in genetic assays.

### MMC^R^ associated with RpmG expression required the presence of MutM

**Figure 4:**
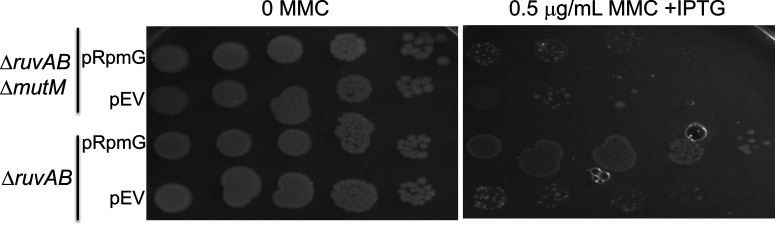
Mitomycin C resistance associated with RpmG expression requires the presence of MutM Viability spot test of MMC^R^ from expressing RpmG (pRpmG) in Δ*ruvAB* cells compared with cells Δ*ruvAB* Δ*mutM*, as indicated.

RpmG is conserved widely across bacterial species, encoded within an operon of conserved gene order *yicR*-*rpmB*-*rpmG*-*mutM*. In *E. coli* the operon is transcribed into least three mRNAs, possibly regulated by *creBC* [27,28]. YicR, formerly called RadC [29], is a putative JAMM-family deubiquitinating (DUB) enzyme [30], and MutM is a DNA glycosylase required for BER [31]. RpmG is a 53 amino acid protein that is frequently annotated in online databases as ribosomal protein L33, although its deletion in *E. coli* had no discernible effect on ribosome function [32]. We investigated if deleting any of *rpmB, yicR and mutM* affected pRpmG induced MMC^R^ in Δ*ruvAB* cells. MMC^R^ associated with expression of pRpmG was lost in Δ*mutM* cells (Figure 4), but deletions of *yicR* or *rpmB* had no effect. Therefore MMC^R^ associated with RpmG is functionally dependent on MutM. We were unable to identify any single or combined amino acid substitutions in RpmG that abolished MMC^R^ (summarised in supplementary material). This is possibly because RpmG has a non-catalytic role that facilitates MutM activity through physical interaction involving multiple amino acids.

## DISCUSSION

ASKA libraries have been used to identify genetic factors that influence phenotypes in bacteria [33–35]. We used one version of this resource [25] to screen for novel *E. coli* genes involved in DNA repair, indicated by a MMC resistance (MMC^R^) phenotype. MMC is a potent genotoxic agent by forming inter- and intra-strand cross-links in DNA that block replication and transcription and lead to cell death unless the lesion is repaired. One way to repair MMC lesions is through homologous recombination, which in *E. coli* can involve a ‘resolvasome’ complex called RuvABC. The importance of RuvABC in MMC repair is exemplified by the MMC sensitivity of cells lacking RuvABC (e.g. Δ*ruvAB* in Figure 1A). MMC^R^ from expression of *ygaQ* was evident in both a Δ*ruvAB* or Δ*ruvC*  strain. This is consistent with YgaQ acting aside from Holliday junction processing by RuvABC, but deletion of YgaQ alone had no MMC sensitivity phenotype, suggesting that at least in the growth conditions we used, YgaQ is subservient to RuvABC. We speculate that actions of YgaQ in DNA repair or coping with genotoxic stress may become apparent only in response to specific stresses. An interesting observation made from combining Δ*ruvAB* with other gene deletions was that the MMC^R^ phenotype from YgaQ expression was lost when cells also lacked UvrD. This suggests that in *E. coli* lacking RuvABC and alternative system for dealing with MMC requires either combined actions of UvrD with YgaQ, or that YgaQ can promote recovery assisted by UvrD. UvrD is a facilitator of DNA repair by exposing lesions for further processing, in some cases by generating ssDNA for removal by nucleases [36]. We observed nuclease activity of YgaQ directed to 5′-ended ssDNA, which could therefore potentially degrade ssDNA generated from the 3′ to 5′ translocation polarity of UvrD. Nuclease activity of YgaQ was abolished or much reduced by introducing several amino acid substitutions (generating YgaQ^DM^), but not by individual amino acid substitutions. Database analyses of the W3110 strain 110 amino acid protein gave significant homology to α-amylases, which hydrolyse oligosaccharides into their constituent sugars. It may be plausible that in YgaQ the same kind of fold could be utilized for binding to the DNA backbone leading to hydrolysis of phosphodiester bonds requiring DNA binding and active site chemistry of aspartate, glutamate and arginine residues that were mutated in YgaQ^DM^.

Observation of a role for RpmG in repair of DNA lesions in *E. coli* resembles the reported extra-ribosomal functions of ‘ribosomal’ proteins in prokaryotes and eukaryotes, including in DNA repair [37,38]. One such ‘idiosyncratic’ ribosomal protein physically interacts with a eukaryotic DNA repair enzyme, stimulating its activity [39,40]. It is possible that RpmG protein may act in a similar way with MutM.

## METHODS

### Processing the ASKA library for plasmid DNA

The ASKA library of plasmid encoded hexa-histidine tagged *E. coli* W3110 proteins was obtained from NBRP-*E. coli* at NIG (http://www.shigen.nig.ac.jp/ecoli/strain/top/top.jsp), described in [25]. The library contains 4364 open reading frames cloned individually into a plasmid vector for IPTG induced protein expression. Agar plates of up to 96 colonies were flooded with 3–5 ml of sterile LB broth and this was used to extract plasmid DNA by standard methods into 100 μl of sterile water, thus generating sub-sections of the library, each containing up to 96 different *E. coli* W3110 genes. For the present study, eight undergraduate students were each given aliquots of either five or six sub-sections of the library for transformation into *E. coli* MG1655 Δ*ruvAB* to begin the screening process, described below and in Figure 1(B).

### Strains and plasmids

Details of the *E. coli* strains and plasmids used in this work are given in Supplementary Tables S1 and S2.

### Viability spot tests and P1 transductions

For viability spot tests of *E. coli* growths, LB cultures were grown to attenuance of 0.4 measured at 600 nm, and then serially diluted 10-fold into M9 salts as indicated in the figures. Typically, 15 μl of diluted cells was spotted on to appropriate LB agar.

Transductions were used to move around antibiotic resistance gene deletion cassettes using standard methods from P1 lysates, briefly: an overnight culture of the strain to be transduced was inoculated into 8 ml fresh broth and grown to attenuance of 0.8. Pelleted cells were resuspended for 10 min at ambient temperature in 1 ml buffer MC (100 mM MgSO_4_, 5 mM CaCl_2_), prior to addition of P1 lysate of various titers prepared to contain the desired selectable gene cassette. Incubation, at typically 37°C for 30 min, was followed by addition of sodium citrate to 1 mM, followed by suspension of the P1–*E. coli* mixture in warm liquid 0.6% agar broth and plating on to agar containing the appropriate antibiotic selection. Incubation was at 37°C for up to 48 h to allow growth of resistant colonies that were then purified by antibiotic selection and verified for correct insertion of the desired gene cassette.

### Screening ASKA plasmids for mitomycin C resistance in *E. coli* Δ*ruvAB* cells

This is summarized in Figure 1(B). A group of eight undergraduate research project students transformed an apramycin resistant Δ*ruvAB E. coli* strain N6029 (Supplementary Table S1) with ASKA plasmids and plated cells on to chloramphenicol (15 μg/ml) LB agar. In total approximately 11000 of the resultant colonies were master gridded on to LB agar containing chloramphenicol (15 μg/ml), and then replica plated on to LB agar containing either chloramphenicol (15 μg/ml), or chloramphenicol plus MMC (0.2 μg/ml) and IPTG (0.5 mM). A positive control plasmid that gives MMC^R^ in Δ*ruvAB E. coli* by expressing the resolvase Hjc [17] was included in every stage to compare to ASKA clones. Note that using Δ*ruvAB* cells for screening MMC^R^ from the ASKA library was appropriate because *ruvA* and *ruvB* genes encoding the RuvAB complex (RuvA_4 or 8_-RuvB_12_), were on separate ASKA 96-well plates, removing the potential for false-positive MMC^R^ that could arise if from *ruvA* and *ruvB* were encoded on the same plasmid.

### Mutagenesis of *ygaQ* and purification of *E. coli* YgaQ and YgaQ^DM^ proteins

The ASKA plasmid containing *ygaQ* was mutagenized using the Q5 Base-Changer strategy from New England Biolabs. Primer sequences can be provided on request. For protein analysis the gene encoding *E. coli* W3110 YgaQ was synthesized using GeneArt (Life Technologies), to include restriction sites for sub-cloning and optimization for codon usage. Sub-cloning of *ygaQ* into pET14b facilitated expression of N-terminally hexa-histidine tagged YgaQ. The same GeneArt process was used to synthesize the gene encoding YgaQ^DM^, with appropriate nucleotide substitutions for the following amino acid substitutions: D23G, D25G, E107G, R108S and K109STOP. YgaQ and YgaQ^DM^ were over-expressed and purified in the same way: Briefly, strain BL21 AI harbouring the desired plasmid was induced with arabinose at 37°C for 4 h. Cells lysed in buffer (20 mM Tris·HCl pH 8.0, 500 mM NaCl, 10% (v/v) glycerol, 10 mM imidazole) were passed into a 5 ml Hi-Prep nickel chelation column, with YgaQ proteins luting within a gradient of 0–250 mM imidazole. Fractions containing YgaQ were pooled, dialysed into a new buffer (20 mM Tris pH 8.0, 100 mM NaCl, 1 mM DTT, 10% (v/v) glycerol) and passed into a 5 ml Hi-Trap heparin column, to which YgaQ proteins did not bind but were collected in the flow-through.

### DNA assays

Base sequences of DNA strands used to construct substrates are given in Supplementary materials. DNA strands were custom synthesized and HPLC purified by Sigma–Aldrich. DNA strands (300 ng) were ^32^P labelled at their 5′ ends by incubation with T4 polynucleotide kinase (PNK) and **γ^32^P-ATP (1 h, 37°C) followed by heat inactivation of PNK. Unincorporated ATP was removed from these reactions using Bio-Spin 6 columns (Bio-Rad). Resulting end-labelled DNA was annealed to other unlabelled DNA strands (900 ng) in buffer (150 mM sodium chloride and 15 mM sodium citrate, pH 7.0) by heating to 95°C for 2 min followed by gradual cooling to room temperature. DNA substrates were then purified, to remove un-annealed oligonucleotide or incomplete DNA structures, by electrophoresis through a 10% acrylamide Tris–borate–EDTA (TBE) gel followed by autoradiography, excision of gel slice and elution by diffusion at 4°C into 250–500 μl of 10 mM Tris·HCl, 50 mM sodium chloride pH 7.5. Nuclease assays were in buffer HB (7 mM Tris·HCl pH 8.0, 9% glycerol, 50 mM NaCl, 100 μg/ml BSA) supplemented with 10 mM magnesium chloride at 37°C for 10 min. Reactions were stopped by addition of 1 mg/ml proteinase K, 2.5% w/v SDS prior to electrophoresis through 10% TBE–acrylamide gels 1×TBE buffer.

## Abbreviations

ASKA: A Complete Set of *Escherichia coli* K-12 ORF Archive
BER: base excision repair
DUB: deubiquitinating
MMC: mitomycin C
MMC^R^: mitomycin C resistance
PNK: polynucleotide kinase
TBE: Tris–borate–EDTA
